# Limbic encephalitis associated with anti-NH_2_-terminal of α-enolase antibodies: A clinical subtype of Hashimoto encephalopathy

**DOI:** 10.1097/MD.0000000000006181

**Published:** 2017-03-10

**Authors:** Toru Kishitani, Akiko Matsunaga, Masamichi Ikawa, Kouji Hayashi, Osamu Yamamura, Tadanori Hamano, Osamu Watanabe, Keiko Tanaka, Yasunari Nakamoto, Makoto Yoneda

**Affiliations:** a The Second Department of Internal Medicine, Faculty of Medical Sciences, University of Fukui, Fukui; b Department of Neurology and Geriatrics, Kagoshima University Graduate School of Medical and Dental Sciences, Kagoshima; c Department of Neurology, Kanazawa Medical University, Ishikawa; d Faculty of Nursing and Social Welfare Sciences, Fukui Prefectural University, Fukui, Japan.

**Keywords:** anti-NH_2_-terminal of α-enolase antibodies, autoimmune limbic encephalitis, Hashimoto encephalopathy, immunotherapy

## Abstract

Several types of autoantibodies have been reported in autoimmune limbic encephalitis (LE), such as antibodies against the voltage-gated potassium channel (VGKC) complex including leucine-rich glioma inactivated 1 (LGI1). We recently reported a patient with autoimmune LE and serum anti-NH_2_-terminal of α-enolase (NAE) antibodies, a specific diagnostic marker for Hashimoto encephalopathy (HE), who was diagnosed with HE based on the presence of antithyroid antibodies and responsiveness to immunotherapy. This case suggests that LE patients with antibodies to both the thyroid and NAE could be diagnosed with HE and respond to immunotherapy. The aim of this study was to clarify the clinicoimmunological features and efficacy of immunotherapy in LE associated with anti-NAE antibodies to determine whether the LE is a clinical subtype of HE.

We examined serum anti-NAE antibodies in 78 LE patients with limbic abnormality on magnetic resonance imaging and suspected HE based on positivity for antithyroid antibodies. Nineteen of the 78 patients had anti-NAE antibodies; however, 5 were excluded because they were double positive for antibodies to the VGKC complex including LGI1. No antibodies against the *N*-methyl-D-aspartate receptor (NMDAR), contactin-associated protein 2 (Caspr2), γ-aminobutyric acid-B receptor (GABA_B_R), or α-amino-3-hydroxy-5-methylisoxazole-4-propionic acid receptor (AMPAR) were detected in the 19 patients. Among the remaining 14 who were positive only for anti-NAE antibodies, the median age was 62.5 (20–83) years, 9 (64%) were women, and 8 (57%) showed acute onset, with less than 2 weeks between onset and admission. Consciousness disturbance (71%) and memory disturbance (64%) were frequently observed, followed by psychiatric symptoms (50%) and seizures (43%). The frequency of these symptoms significantly differed between the acute- and subacute-onset groups. Abnormalities in cerebrospinal fluid and electroencephalogram were commonly observed (92% for both). Tumors were not identified in any cases. All patients responded to immunotherapy or spontaneously remitted, thereby fulfilling the criteria of HE.

This study demonstrated that LE associated with anti-NAE antibodies is a nonparaneoplastic LE and various limbic symptoms that depend on the onset type. Favorable therapeutic efficacy suggests that this LE can be considered a clinical subtype of HE and that anti-NAE antibodies may be a promising indicator of the need for immunotherapy.

## Abstract 2

Supplemental Digital Content is available in the text

## 1 Introduction

Limbic encephalitis (LE) is one of the most common forms of encephalitis and predominantly affects the limbic system.^[1]^ Consciousness disturbance, psychiatric symptoms, and memory disturbance are common symptoms in patients with LE. Excluding herpes virus infection, autoimmune mechanisms account for the majority of the pathogenesis of LE.^[2,3]^ Indeed, autoantibodies against various antigens, such as the *N*-methyl-D-aspartate receptor (NMDAR), voltage-gated potassium channel (VGKC) complex that includes leucine-rich glioma inactivated 1 (LGI1) and contactin-associated protein 2 (Caspr2), γ-aminobutyric acid-B receptor (GABA_B_R), and α-amino-3-hydroxy-5-methylisoxazole-4-propionic acid receptor 1 and 2 (AMPAR1/2), have been identified in autoimmune LE, especially in paraneoplastic LE associated with neoplasms.^[4–9]^ The identification of antibodies would not only help clarify the pathogenesis of LE but would also be useful for the diagnosis and prognostic predictions of LE because both the clinical features and the efficacy of LE therapies are generally related to the type of antibodies.^[3,9,10]^ However, there are still many autoimmune forms of LE, especially nonparaneoplastic LE, for which the related antibodies remain unidentified.

We previously discovered autoantibodies against the NH_2_-terminal of α-enolase (NAE) in the serum of patients with Hashimoto encephalopathy (HE) using proteomic analyses and demonstrated the high specificity of these antibodies for HE.^[11,12]^ HE is an autoimmune encephalopathy associated with Hashimoto thyroiditis, also known as steroid-responsive encephalopathy associated with autoimmune thyroiditis.^[13–16]^ Our recent case report described a patient with autoimmune LE and serum anti-NAE antibodies who was diagnosed with HE based on positive serum antithyroid antibodies and responsiveness to immunotherapy,^[17]^ suggesting that patients with LE and anti-NAE antibodies could respond to immunotherapy and that LE associated with anti-NAE antibodies may be a clinical subtype of HE.

In this study, we conducted clinical analyses of a large number of patients with LE and anti-NAE antibodies who were suspected of having HE to clarify the clinical and immunological features of LE associated with anti-NAE antibodies. We also sought to evaluate the clinical utility of anti-NAE antibodies in LE by analyzing the therapeutic efficacy of immunotherapy to determine whether LE associated with anti-NAE antibodies is a type of HE.

## 2 Materials and methods

### 2.1 Subjects

We investigated the presence of anti-NAE antibodies in sera from 78 patients who presented with limbic symptoms accompanied by abnormal signals in limbic areas on magnetic resonance imaging (MRI) and in whom HE was suspected because of positivity for serum antithyroid antibodies (antibodies to thyroglobulin and/or thyroid peroxidase).^[14]^ These patients were examined at the University of Fukui, or the patient's serum and clinical information were referred from other institutions and examined for anti-NAE antibodies between 2005 and 2014. In all cases, infectious encephalitis was excluded due to a negative for bacterial and fungal culture and the presence of DNA from herpes simplex, cytomegalo-, varicella zoster, and Epstein–Barr viruses in the cerebrospinal fluid (CSF). If anti-NAE antibodies were detected, the serum was further examined for antibodies to the NMDAR, VGKC complex, LGI1, Caspr2, GABA_B_R, and AMPAR1/2 to exclude LE associated with these antibodies. The methods used to detect these antibodies are described below.

The ethics committees of the University of Fukui, Fukui Prefectural University, Kanazawa Medical University, and Kagoshima University approved this research, and written permission was obtained from each subject.

### 2.2 Investigation of clinical features

We investigated the clinical features of the patients with LE and anti-NAE antibodies, including LE-related symptoms at admission, laboratory findings, and the therapeutic efficacy of treatment, using written questionnaires that were completed by the authors or the referring neurologists who examined and treated each patient. The patients were further categorized into 2 groups according to the time from symptom onset to admission: the acute-onset group, with a time of less than 2 weeks; and the subacute-onset group, with a time of over 2 weeks to several months. Thyroid function was evaluated based on serum levels of thyroid stimulating hormone, free thyroxine (T4), and free triiodothyronine (T3). Cell counts and protein concentrations in CSF as well as electroencephalograms were evaluated before treatment. All patients underwent tumor screening, including CT, MRI, and/or positron emission tomography with [^18^F]fludeoxyglucose. Neurological outcome was used as a measure of the efficacy of immunological treatments and was evaluated with the modified Rankin Scale (mRS) as follows: full recovery from encephalitis, which corresponds to an mRS score of 0 or 1; good recovery with mild residual disability, consistent with an mRS score of 2; and limited recovery with moderate or severe residual disability, which corresponds to an mRS score of more than 3. All patients with LE and anti-NAE antibodies were followed up to monitor relapse.

### 2.3 Detection of anti-NAE antibodies

**Figure 1:**
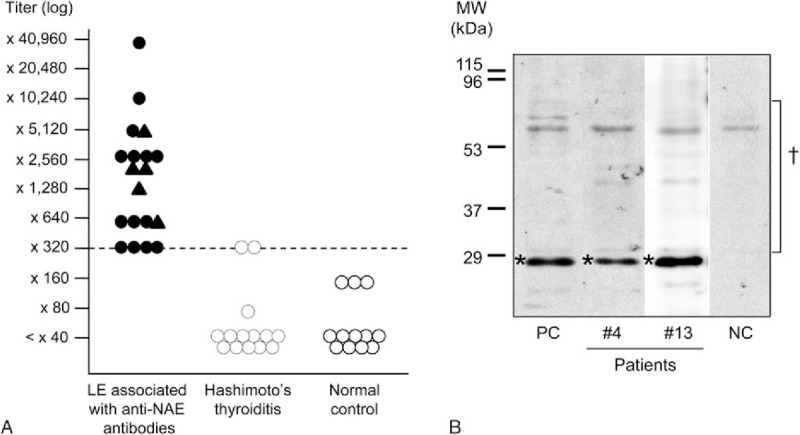
Titer distributions and immunoblotting of anti-NH_2_-terminal of α-enolase (NAE) antibodies. A, Anti-NAE antibody titers of patients with LE associated with anti-NAE antibodies, patients with Hashimoto thyroiditis, and normal controls. The cut-off level for positivity is indicated by the dashed line (titer: ×320). In patients with LE associated with anti-NAE antibodies, triangles indicate patients with antibodies to both NAE and other antigens (i.e., the voltage-gated potassium channel (VGKC) complex); circles indicate patients who are positive only for anti-NAE antibodies. B, Representative findings of immunoblotting of a recombinant amino NAE with serum from patients with limbic encephalitis (LE) associated with anti-NAE antibodies. An NAE signal was detected around 29 kDa for the sera of Cases 4 and 13. ∗Position of the recombinant NAE; ^†^Derivatives of human cells that were cultured for NAE expression and showed nonspecific reactions with sera; NC = negative control, PC = positive control.

A recombinant NAE expressed in cultured human embryonic kidney (HEK) 293 cells was purified through a His column (ProBond Protein Purification kit, Invitrogen, CA). An immunoblotting analysis of serum antibodies against the recombinant NAE was performed using a gel electrophoresis system (BE-220, BIO CRAFT, Tokyo, Japan) and 12% sodium lauryl sulfate-polyacrylamide gel electrophoresis, as previously described.^[11,12]^ The proteins on the gel were blotted onto a polyvinylidene difluoride membrane (Hybond-P, GE Healthcare UK Ltd., Buckinghamshire, UK) with a blotting apparatus (KS-8453, Oriental Instrument, Tokyo, Japan) at 0.3 mA/cm^2^ for 8 hours at 4°C. To detect the band specific to the NAE, serum was applied to the membrane, which was then incubated in 1% gelatin for 1 hour at room temperature. Horseradish peroxidase-conjugated anti-human goat IgG Fc (ICN Pharmaceuticals Inc, Costa Mesa, CA) was then applied to the membrane as the secondary antibody, and the membranes were fluorescently labeled and developed on X-ray films (BioMax, Kodak, NY) or using the ImageQuant LAS4000 mini (GE healthcare, Tokyo, Japan). In addition to the serum samples from the patients, the serum of 12 healthy individuals served as control samples. Serum samples from 14 patients with Hashimoto thyroiditis who did not present with any neuropsychiatric symptoms were also investigated. The titer of anti-NAE antibodies was defined as the highest dilution of the patient's serum that was positive for the antibodies. The cut-off titer was set at 1/320 because the titers of the normal control samples and those from patients with Hashimoto thyroiditis were below 1/160 and 1/320, respectively (Fig. 1A).

### 2.4 Detection of anti-NMDAR antibodies

To exclude anti-NMDAR encephalitis, antibodies against the NMDAR were examined according to previously reported methods.^[18]^ Briefly, cDNAs encoding NR1 (GluN1) and NR2B (GluN2B) were ligated into expression vectors and transfected into HEK 293 cells. These cells were incubated with patient sera (1:40) overnight at 4°C and then with FITC-conjugated rabbit antihuman IgG (BD Biosciences, Tokyo, Japan). The staining was observed under a fluorescence microscope.

### 2.5 Detection of anti-VGKC complex antibodies

To exclude encephalitis associated with anti-VGKC complex antibodies, these antibodies were examined according to methods that have been reported elsewhere.^[19,20]^ Briefly, antibodies to the VGKC were measured by immunoprecipitation of ^125^I-dendrotoxin-labeled VGKCs (this method labels types kv1.1, 1.2, and 1.6). Indirect immunohistochemistry was performed with serum that was diluted 1:200 to 1:800 on frozen, paraformaldehyde-fixed, saponin-permeabilized rat brain tissue (preperfused in phosphate-buffered saline).

### 2.6 Detection of antibodies against the LGI1, Caspr2, GABA_B_R, and AMPAR1/2

To exclude encephalitis associated with antibodies against the LGI1, Caspr2, GABA_B_R, and AMPAR1/2, these antibodies were examined using a cell-based assay kit (Autoimmune Encephalitis Mosaic 1, EUROIMMUN, Luebeck, Germany).^[21]^ Anti-NMDAR antibodies were also examined using this kit to confirm the reliability of this assay system through comparison with the results from the conventional method described above. The test kit is based on an indirect immunofluorescence technique and uses transfected HEK 293 cell lines that recombinantly express the target antigens (i.e., NMDAR (subunit NR1), AMPAR1/2 (GluR1/GluR2), GABA_B_R (subunit B1), LGI1 (membrane-bound fusion protein), and Caspr2). The recombinant cells are acetone-fixed on a glass slide as millimeter-sized fragments (BIOCHIPs).

Diluted patient's serum (1:10) was incubated with the antigens expressed on the BIOCHIP slide for 30 minutes at room temperature. Then, the slide was incubated with fluorescein-labeled antihuman antibodies (FITC-conjugated antihuman IgG [goat]) for 30 minutes at room temperature. Fluorescence was examined using a fluorescence microscope (OLYMPUS BX53; Olympus, Tokyo, Japan).

### 2.7 Statistical analysis

Fisher exact test was used to compare proportions between groups. The mRS scores before and after treatment were compared by Wilcoxon signed rank test, while the mRS scores between groups were compared by Mann–Whitney *U* test. All of the statistical analyses were performed using SPSS Statistics ver. 17.0 (SPSS Japan Inc, Tokyo, Japan), and *P* < 0.05 was considered significant.

## 3 Results

### 3.1 Antibodies against the NAE, NMDAR, VGKC complex, LGI1, Caspr2, GABA_B_R, and AMPAR1/2

We detected anti-NAE antibodies in the serum of 19 of the 78 (24%) patients with LE and suspected HE. Figure 1B shows representative immunoblotting findings for a recombinant amino NAE with patient sera (Cases 4 and 13). The anti-NAE antibody titers of the patients ranged from 1/320 to 1/40,960 (Table 1, Fig. 1A). Anti-NMDAR antibodies were not detected in any of the 19 patients during either of the examinations using the conventional methods and the cell-based assay kit, suggesting the reliability of the assay kit. Antibodies to the Caspr2, GABA_B_R, and AMPAR1/2 were also not detected in any of the 19 patients. In contrast, 5 patients (26%) were positive for anti-VGKC complex antibodies in addition to anti-NAE antibodies. Four of the 5 patients who had anti-VGKC complex antibodies were also positive for anti-LGI1 antibodies.

**Table 1:**
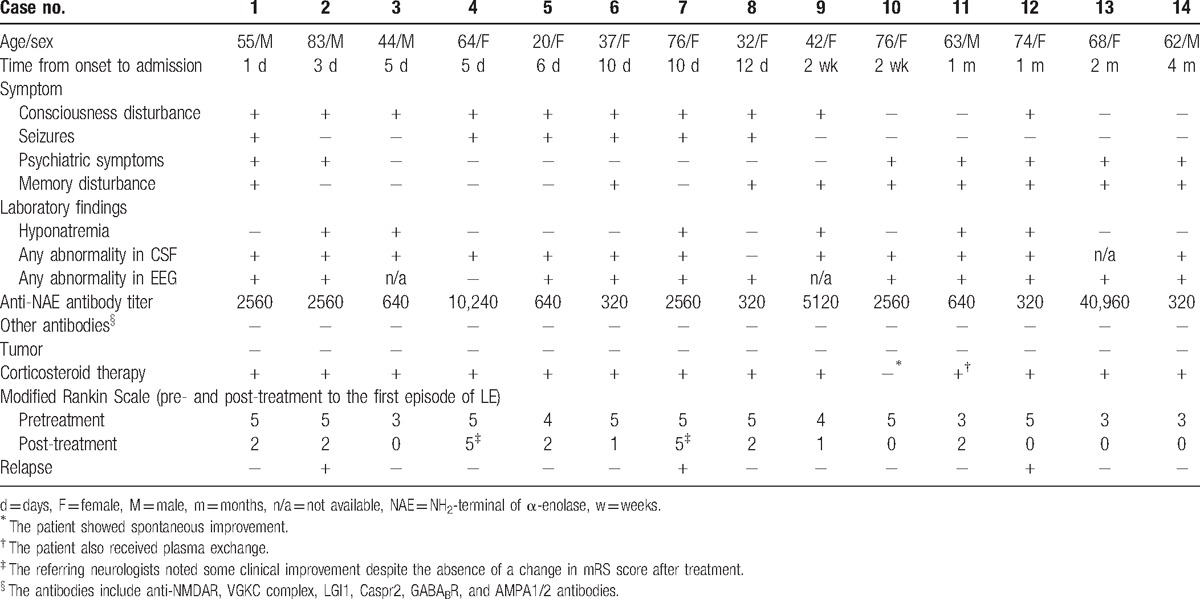
Demographic and clinical features of patients with limbic encephalitis associated with anti-NAE antibodies.

### 3.2 Clinical features

To investigate the clinical findings of LE associated with anti-NAE antibodies, we excluded the 5 patients who were double-positive for anti-VGKC complex antibodies including anti-LGI1 antibodies because these additional antibodies could affect the clinical findings in these patients. The clinical features and outcomes of the remaining 14 patients who were positive only for anti-NAE antibodies are displayed in Tables 1 and 2, while those for the 5 double-positive patients are shown in Supplemental Table 1.

**Table 2:**
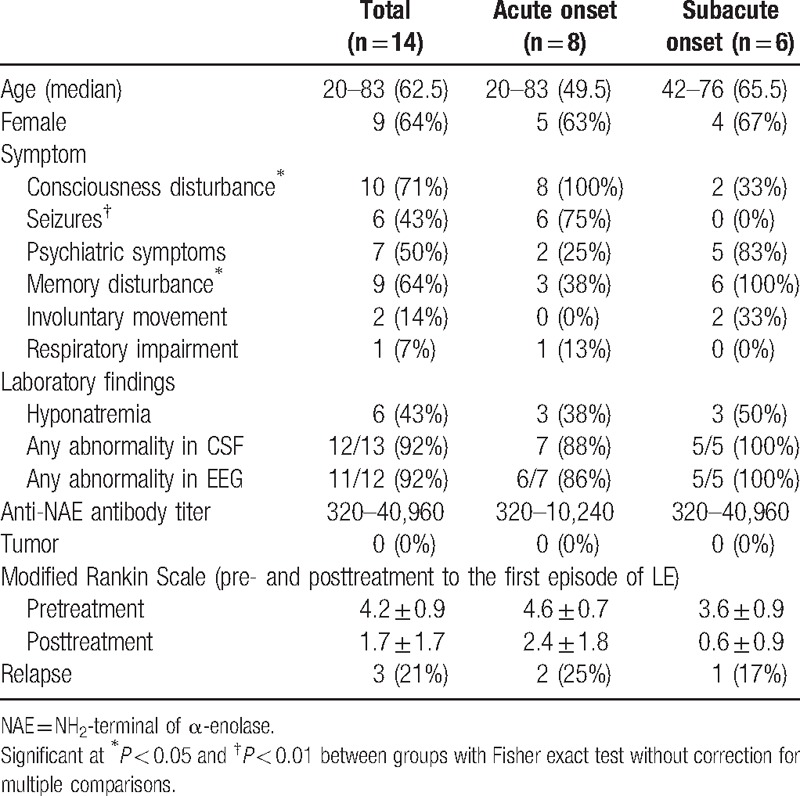
Demographic and clinical features of patients with limbic encephalitis associated with anti-NAE antibodies, classified into onset type.

The patients consisted of 5 men and 9 women, with a median age 62.5 years (range: 20–83). The duration between onset and admission varied, with a range from 1 day to 4 months. Eight of the 14 (57%) patients showed an acute onset of less than 2 weeks (Cases 1–8), whereas the other 6 patients showed a subacute onset of within 2 weeks to 4 months (Cases 9–14). Three patients had a history of Hashimoto thyroiditis and had been adequately treated.

Among the 14 patients, consciousness disturbance (71%) and memory disturbance (64%) were frequently observed, followed by psychiatric symptoms (50%) and seizures (43%) (Table 2). Psychiatric symptoms included hallucinations, delusions, mood changes, agitation, and abnormal behavior. All seizures observed in the patients were generalized convulsions. Involuntary movements and respiratory impairment were rare (14% and 7%, respectively). Interestingly, the frequencies of consciousness disturbance and seizures were significantly higher in the acute-onset group (100% and 75%, respectively) than in the subacute-onset group (33% and 0%, respectively) (*P* < 0.05 and < 0.01, respectively). In contrast, psychiatric symptoms and memory disturbance were more frequently observed in the subacute-onset group (83% and 100%, respectively) than in the acute-onset group (25% and 38%, respectively) (*P* = 0.10 and <0.05, respectively). These findings suggest that symptoms of LE associated with anti-NAE antibodies are not uniform and may differ depending on onset type.

**Figure 2:**
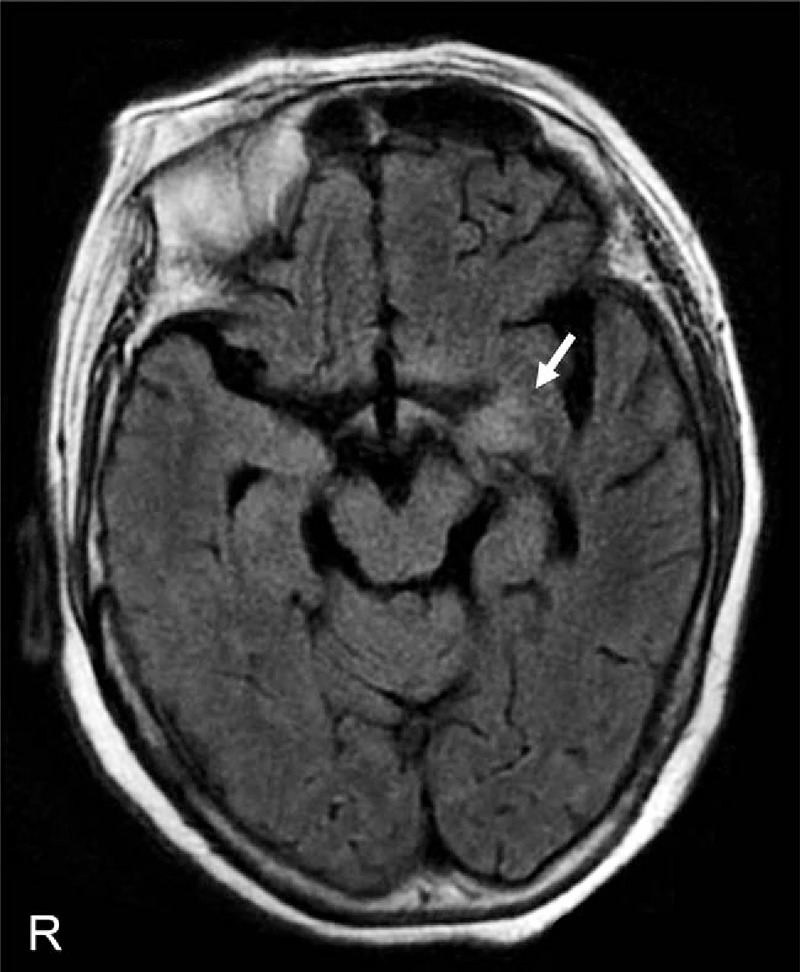
Brain MRI findings of a representative patient (Case 12). Fluid-attenuated inversion recovery images show abnormal hyperintense signals in the left medial temporal lobe (arrow). MRI = magnetic resonance imaging.

Regarding thyroid function, all 14 patients were clinically euthyroid except for 1 patient with low T4 who presented with no symptoms related to hypothyroidism. Hyponatremia, defined as a serum sodium concentration of less than 135 mEq/L, which is frequently observed in LE associated with anti-LGI1 antibodies, was observed in 6 (43%) patients at admission (Table 2). When the CSF was examined, some type of abnormality was observed in 12 of 13 patients (92%). The cell counts and protein concentrations in the CSF ranged from 0 to 333/mm^3^ and from 18 to 317 mg/dL, respectively. Five patients who were double-positive for anti-VGKC complex antibodies, including anti-LGI1 antibodies, showed less frequent in CSF abnormalities than 14 patients who were positive only for anti-NAE antibodies (*P* < 0.05) (Supplemental Table 1). On MRI, abnormal signals were observed in the unilateral or bilateral medial temporal lobes of all patients, as defined by the selection criteria of this study (Fig. 2). On electroencephalograms, diffuse slow wave activity with epileptic discharges was found in 11 of 12 patients (92%), although most patients were clinically euthyroid.

All patients were treated with immunotherapy except for 1 patient with spontaneous improvement (Case 10). All of the treated patients responded to immunotherapy at the first episode of LE, thereby fulfilling the diagnostic criteria of HE: neuropsychiatric symptoms, positive for antithyroid antibodies, and responsiveness to immunotherapy.^[13–15]^ More specifically, 11 of the 13 (85%) treated patients showed full or good recovery (posttreatment mRS score of 0 to 2). Severe symptoms (mRS score of 5) persisted after treatment in the remaining 2 patients (Cases 4 and 7), but the referring neurologists reported slight improvement of consciousness in both patients. The mRS scores after treatment (1.7 ± 1.7, mean ± SD) were significantly decreased compared with those before treatment (4.2 ± 0.9) in the 13 treated patients (*P* < 0.005). The patients with some residual disability (mRS score of more than 2) more frequently presented with acute onset and seizures (86% and 71%, respectively) than the patients who fully recovered (mRS score of 0 or 1) (29% and 14%, respectively) (*P* = 0.10 for both) (Supplemental Table 2). No significant difference in the mean pretreatment mRS score was found between the full-recovery group (4.0 ± 1.0) and the residual-disability group (4.6 ± 0.8), suggesting that outcomes may not depend on symptom severity at admission. Methylprednisolone pulse therapy (1000 mg/d for 3 days) was given to all treated patients, and 9 patients were treated with methylprednisolone pulse therapy followed by oral prednisolone therapy (median starting dose of prednisolone equal to 50 mg/d). One patient was additionally treated with plasma exchange (Case 11) due to a poor response to methylprednisolone pulse therapy. The patient responded well to this additional therapy.

Neither the pre- nor the posttreatment mRS score was associated with the titer of anti-NAE antibodies. In addition, in 3 patients for whom paired serum samples were available (Cases 11, 12, and 14; the intervals were 3.2, 6.1, and 5.5 years, respectively), the anti-NAE antibodies remained positive after treatment. The initial titers were relatively low in these patients (×640 for Case 11 and × 320 for Cases 12 and 14).

The median follow-up period was 33 months (range: 3–100 months). No tumors were identified in any cases, either at admission or during the follow-up period. Three patients (21%), 1 of which was in the full-recovery group, relapsed during the follow-up period despite consecutive oral prednisolone therapy. The time period between initial onset and relapse ranged from 1 to 4 months. Although 1 of the 3 patients died of pneumonia during treatment of the relapse, the remaining 2 recovered from the relapse after additional corticosteroid therapy.

## 4 Discussion

In this study, we demonstrated the clinical and immunological features of LE associated with anti-NAE antibodies. The typical clinical picture involves nonparaneoplastic LE with various limbic symptoms that differ depending on onset type in middle-aged to senior females with antithyroid antibodies (Tables 1, 2). Importantly, all of the patients in this study responded to immunotherapy except for 1 patient who spontaneously recovered, suggesting that anti-NAE antibodies may be a promising biomarker that can predict favorable therapeutic efficacy for LE. Good responsiveness to immunotherapy with positive antithyroid antibodies also indicates that LE associated with anti-NAE antibodies is a clinical subtype of HE.

HE is characterized as immunotherapy-responsive encephalitis with various neuropsychiatric symptoms and antithyroid antibodies.^[12–16]^ Various symptoms are used to define clinical subtypes of HE, such as the Creutzfeldt–Jakob disease-like form and the cerebellar ataxic form.^[22–24]^ However, the diversity of symptoms and the high prevalence of antithyroid antibodies in healthy populations may make the diagnosis of HE difficult. We discovered anti-NAE antibodies, autoantibodies that specifically recognize only the NH_2_-terminal of α-enolase, in the serum of patients with HE by proteomic analyses and demonstrated a high prevalence and specificity of these antibodies in patients with HE.^[11,12]^ In contrast, neither the COOH-terminal, mid-region, nor whole structure of α-enolase showed specificity for HE.^[11]^ Because the α-enolase is a ubiquitous glycolytic enzyme that is abundantly expressed in the cytoplasm of most tissues,^[25,26]^ antibodies to the whole α-enolase structure showed no specificity, as reported in various autoimmune disorders, such as systemic lupus erythematosus, multiple sclerosis, and rheumatoid arthritis.^[26]^ The COOH-terminal portion of α-enolase also nonspecifically reacted with serum collected from individuals with Hashimoto thyroiditis, from normal individuals, and from those with HE. The high specificity of antibodies to the NH_2_-terminal of α-enolase (i.e., NAE) for HE suggests that the NH_2_-terminal displays specific immunogenicity and that anti-NAE antibodies serve as a diagnostic biomarker of HE.^[11,12]^ In fact, the patients with LE and anti-NAE antibodies in this study met the criteria for HE due to their responsiveness to immunotherapy in addition to the presence of neuropsychiatric symptoms and antithyroid antibodies. Thus, LE associated with anti-NAE antibodies would be included as a type of HE.

To identify the clinical features of LE associated with anti-NAE antibodies, we compared the features of this form of LE with those of other representative autoimmune forms of LE or encephalitis from the literature, such as LE associated with antibodies to the LGI1, AMPAR, or GABA_B_R, and encephalitis associated with anti-NMDAR antibodies (Table 3).^[4,5,7,8]^ The principal difference between LE associated with anti-NAE antibodies and these other forms of LE or encephalitis is whether the disease is tumor related. Most of other forms of LE or encephalitis are related to tumors as part of a paraneoplastic syndrome, but no tumors were identified in the patients with LE associated with anti-NAE antibodies in this study. Although tumors are not always identified even in paraneoplastic LE, the absence of tumors in the patients with LE associated with anti-NAE antibodies indicates that this LE should be suspected in cases of autoimmune LE without tumors or paraneoplastic antibodies. Moreover, involuntary movements and respiratory impairment, which are characteristic of encephalitis associated with anti-NMDAR antibodies,^[4]^ were observed less frequently in cases of LE associated with anti-NAE antibodies. The percentage of abnormality in the CSF was observed more frequently in LE associated with anti-NAE antibodies than in LE associated with anti-LGI1 antibodies.^[5,6]^ A similar difference was obtained from the comparison of cases that were double-positive for anti-VGKC complex antibodies, including anti-LGI1 antibodies, in the present study (Supplemental Table 1). In addition to the presence of anti-NAE antibodies, these clinical and laboratory features would be helpful for distinguishing from NMDAR encephalitis and LE associated with anti-LGI1 antibodies. However, the low tumor detection percentage and relatively higher percentage of patients with hyponatremia that are found in LE associated with anti-LGI1 antibodies are similar to those found in LE associated with anti-NAE antibodies (Table 3).^[5,6]^ Moreover, 4 of the 19 patients in the present study had anti-LGI1 antibodies in addition to anti-NAE antibodies, suggesting that LE associated with antibodies to LGI1 should be excluded in patients with LE and anti-NAE antibodies. Further studies are necessary to clarify the clinical and immunological differences between cases that are single- and double-positive for these antibodies.

**Table 3:**
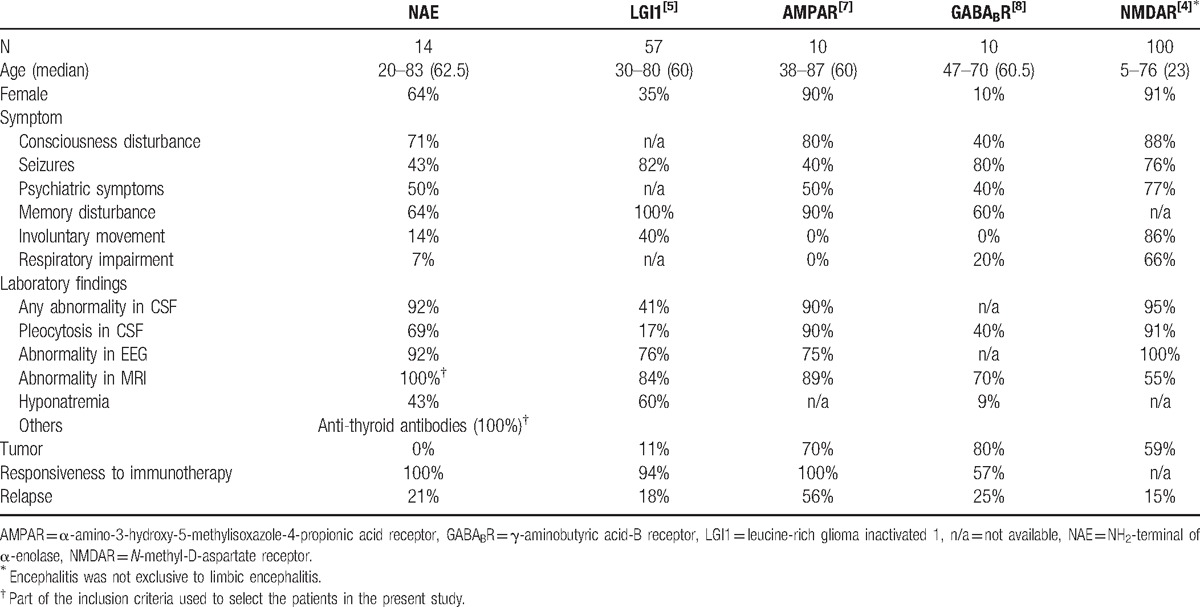
Comparison of clinical features between patients with limbic encephalitis associated with anti-NAE antibodies and those with other types of autoimmune limbic encephalitis.

Unfortunately, the titer of anti-NAE antibodies did not correlate with the clinical severity of HE or with patient outcomes in the present study. However, our previous patch-clamp study showed that CSF containing anti-NAE antibodies obtained from a patient with a cerebellar ataxic form of HE interfered with cerebellar excitatory synaptic transmission,^[27]^ which suggests the involvement of anti-NAE antibodies in the pathogenesis of encephalitis.

The limitations of the present study include the small number of patients and the inclusion of only Japanese patients. Further studies with a large sample size and other races are necessary to confirm our preliminary results.

In conclusion, the present study demonstrated the clinical and immunological features of LE associated with anti-NAE antibodies as a clinical subtype of HE; these features included various limbic symptoms that differed between patients with acute and subacute onset, a lack of accompanying tumors, and favorable responsiveness to immunotherapy. The good therapeutic efficacy suggests that anti-NAE antibodies may indicate the need for immunotherapy. Thus, physicians should more attentively consider the possibility of this form of LE and examine serum anti-NAE antibodies if a patient with LE has antithyroid antibodies without tumors.

## Acknowledgments

The authors thank Tomomi Kame for technical assistance and the physicians who supported this study: Prof Hidenao Sasaki, Dr Moemi Yatsushima, and Dr Masaaki Niino (Hokkaido University); Prof Yasuo Terayama, Dr Yoshinobu Ogino, and Dr Katsuhiro Onodera (Iwate Medical University); Prof Yoshikazu Ugawa and Dr Sachiko Namatame (Fukushima Medical University); Dr Makoto Takahashi and Dr Suguru Matsumoto (Ome Municipal General Hospital); Prof Yuji Wada and Dr Makoto Ishitobi (University of Fukui); Prof Hidekazu Tomimoto, Dr Akihiro Shindo, and Dr Yuichiro Ii (Mie University); Prof Ryosuke Takahashi and Dr Naoto Jingami (Kyoto University); Dr Katsuyoshi Nakajima and Dr Hideo Yagi (Koseikai Takeda Hospital); Dr Yoshiaki Kakehi (Nara City Hospital); Dr Fumiko Kodaka, Dr Makoto Nishii, Dr Sinya Asayama, and Dr Mitsuaki Oki (Kansai Medical University Takii (Hirakata) Hospital); Prof Tatsushi Toda, Dr Yoshihisa Otsuka, Dr Naohiko Seike, and Dr Takehiro Ueda (Kobe University); Dr Kanako Ishihara (Hyogo Prefectural Amagasaki Hospital); Dr Hironobu Magoshi, Dr Yuri Nakamura, and Dr Yasutaka Iwanaga (Matsuyama Red Cross Hospital); Dr Yuki Yanagihara and Dr Naokazu Sasagasako (Fukuoka Higashi Medical Center); and Dr Ken Sonoda, Dr Katsuya Yokoyama, and Dr Hiroto Nakagawa (Kagoshima Medical Association Hospital).

## Supplementary Material

Supplemental Digital Content
