# Supplementary material for: Limbic encephalitis associated with anti-NH2-terminal of α-enolase antibodies: A clinical subtype of Hashimoto encephalopathy
Source: Medicine (Baltimore). 2017 Mar 10;96(10):e6181. doi: 10.1097/MD.0000000000006181 (PMC5348154; doi:10.1097/MD.0000000000006181)
Supplement: Supplemental Digital Content [file medi-96-e6181-s001.docx]

**SUPPLEMENTAL TABLE 1.** Demographic and clinical features of patients with limbic encephalitis who had anti-VGKC complex antibodies in addition to anti-NAE antibodies.

| **Case No.** | **15** | **16** | **17** | **18** | **19** |  | **Total**  **(n = 5)** |
| --- | --- | --- | --- | --- | --- | --- | --- |
| Age | 48 | 69 | 83 | 29 | 62 |  | Median: 62.3 |
| Sex | F | F | F | F | M |  | Female 80% |
| Time from onset to admission | 5 d | 1 m | 1 m | 2 m | 3 m |  | Median: 1.4 m |
| Symptom |  |  |  |  |  |  |  |
| Consciousness disturbance | + | - | - | - | - |  | 1 (20%) |
| Seizures | + | - | + | - | - |  | 2 (40%) |
| Psychiatric symptoms | + | + | - | - | + |  | 2 (40%) |
| Memory disturbance | + | + | + | + | + |  | 5 (100%) |
| Laboratory findings |  |  |  |  |  |  |  |
| Hyponatremia | - | + | n/a | - | n/a |  | 1 (33%) |
| Any abnormality in CSF | - | - | + | - | + |  | 2 (40%)^***^ |
| Any abnormality in EEG | + | + | - | + | - |  | 3 (60%) |
| Anti-NAE antibody titer | 1,280 | 2,560 | 5,120 | 2,560 | 640 |  | 2432 |
| Anti-VGKC complex antibodies | + | + | + | + | + |  | 5 (100%) |
| Anti-LGI1 antibodies | + | + | + | - | + |  | 4 (80%) |
| Other antibodies^**^ | - | - | - | - | - |  | 0 (0%) |
| Tumour | - | - | - | - | - |  | 0 (0%) |
| Corticosteroid therapy | + | + | + | + | +^*^ |  | 5 (100%) |
| Modified Rankin Scale (pre- and post-treatment to the first episode of LE) | | | | | | | |
| Pre-treatment | 4 | 3 | n/a | 2 | 3 |  | Median: 3.7 |
| Post-treatment | 3 | 0 | n/a | 1 | n/a |  | Median: 2.0 |
| Relapse | - | - | - | - | - |  | 0 (0%) |

NAE = NH_2_-terminal of α-enolase, M = male, F = female, d = days, m = months, n/a = not available.

^*^The patient also received IVIg.

^**^The antibodies include anti-NMDAR, Caspr2, GABA_B_R, and AMPAR antibodies.

^***^Significant at *p* < 0.05 between the group of double-positive for anti-VGKC complex and NAE antibodies and that of single-positive for anti-NAE antibodies with Fisher’s exact test without correction for multiple comparisons.

**SUPPLEMENTAL TABLE 2.** Demographic and clinical features of patients with limbic encephalitis associated with anti-NAE antibodies, classified by the neurological outcome

|  | **Patients with fully recovered (n = 7)** | **Patients with residual disability (n = 7)** |
| --- | --- | --- |
| Age (median) | 37-76 (62) | 20-83 (63) |
| Female | 5 (71%) | 4 (57%) |
| Time from onset to admission |  |  |
| Acute (< 2 weeks) | 2 (29%) | 6 (86%) |
| Symptom |  |  |
| Consciousness disturbance | 4 (57%) | 6 (86%) |
| Seizures | 1 (14%) | 5 (71%) |
| Psychiatric symptoms | 4 (57%) | 3 (43%) |
| Memory disturbance | 6 (80%) | 3 (43%) |
| Involuntary movement | 2 (28%) | 0 (0%) |
| Respiratory impairment | 0 (0%) | 1 (14%) |
| Laboratory findings |  |  |
| Hyponatremia | 3 (43%) | 3 (43%) |
| Any abnormality in CSF | 6/6 (100%) | 6 (86%) |
| Any abnormality in EEG | 5/5 (100%) | 6 (86%) |
| Anti-NAE antibody titer | 320 – 40,960 | 320 – 10,240 |
| Tumor | 0 (0%) | 0 (0%) |
| Pre-treatment modified Rankin Scale | 4.0 ｱ 1.0 | 4.6 ｱ 0.8 |
| Relapse | 1 (14%) | 2 (29%) |

NAE = NH_2_-terminal of α-enolase.
